# Retraction: The anti-Alzheimer potential of *Tamarindus indica*: an *in vivo* investigation supported by *in vitro* and *in silico* approaches

**DOI:** 10.1039/d5ra90010g

**Published:** 2025-01-24

**Authors:** Abeer H. Elmaidomy, Usama Ramadan Abdelmohsen, Faisal Alsenani, Hanan F. Aly, Shams Gamal Eldin Shams, Eman A. Younis, Kawkab A. Ahmed, Ahmed M. Sayed, Asmaa I. Owis, Naglaa Afifi, Dalia El Amir

**Affiliations:** a Department of Pharmacognosy, Faculty of Pharmacy, Beni-Suef University Beni-Suef 62514 Egypt; b Department of Pharmacognosy, Faculty of Pharmacy, Minia University Minia 61519 Egypt usama.ramadan@mu.edu.eg; c Department of Pharmacognosy, Faculty of Pharmacy, Deraya University 7 Universities Zone New Minia 61111 Egypt; d Department of Pharmacognosy, Faculty of Pharmacy, Umm Al-Qura University Makkah 21955 Saudi Arabia; e Therapeutic Chemistry Department, National Research Centre (NRC) El-Bouth St., P.O. 12622 Cairo Egypt; f Department of Pathology, Faculty of Veterinary Medicine, Cairo University Giza Egypt; g Department of Pharmacognosy, Faculty of Pharmacy, Nahda University Beni-Suef Egypt; h Department of Pharmacognosy, Faculty of Pharmacy, Heliopolis University for Sustainable Development Cairo Egypt

## Abstract

Retraction of ‘The anti-Alzheimer potential of *Tamarindus indica*: an *in vivo* investigation supported by *in vitro* and *in silico* approaches’ by Abeer H. Elmaidomy *et al.*, *RSC Adv.*, 2022, **12**, 11769–11785, https://doi.org/10.1039/D2RA01340A.

The Royal Society of Chemistry hereby wholly retracts this *RSC Advances* article due to concerns with the reliability of the data.

A number of panels in Fig. 2 and 4 contain partially duplicated sections.

In Fig. 2, part of panel d overlaps with panel f. In Fig. 4 part of panel a overlaps with part of d, part of panel c overlaps with part of panel f, and there is partial overlap between panels b, h and i.

The authors have not been able to provide a suitable explanation on how these duplications occurred.

Given the number and significance of these concerns, the findings presented in this paper are no longer reliable.

This retraction supersedes the information provided in the Expression of concern related to this article.

The authors were informed about the retraction of the article. Usama Ramadan Abdelmohsen, Asmaa I. Owis, Faisal Alsenani, Naglaa Afif, Hanan F. Aly and Abeer H. Elmaidomy have not agreed with the decision, the other authors have not responded.

Signed: Laura Fisher, Executive Editor, *RSC Advances*

Date: 15th January 2025

